# Aerobic exercise affects sleep, psychological wellbeing and immune system parameters among subjects with chronic primary insomnia

**DOI:** 10.4314/ahs.v20i4.29

**Published:** 2020-12

**Authors:** Shehab M Abd El-Kader, Osama H Al-Jiffri

**Affiliations:** 1 Department of Physical Therapy, Faculty of Applied Medical Sciences, King Abdulaziz University, Jeddah, Saudi Arabia; 2 Department of Medical Laboratory Technology, Faculty of Applied Medical Sciences, King Abdulaziz University, Jeddah, Saudi Arabia

**Keywords:** Aerobic exercise, chronic primary insomnia, immune function, sleep quality, psychological wellbeing

## Abstract

**Background:**

Chronic primary insomnia is characterized by long-term difficulties in maintaining and initiating sleep, too early waking up, poor mood, fatigue, impaired concentration and poor quality of life. Exercise training is recommended to prevent and alleviate sleep disorders.

**Objective:**

The aim of the study was to investigate the influence of aerobic exercise training on quality of sleep, psychological wellbeing and immune system among subjects with chronic primary insomnia.

**Material and methods:**

Eighty previously sedentary subjects with chronic primary insomnia subjects enrolled in this study, their age ranged from 35–56 years. All participants were randomly assigned to supervised aerobic exercise intervention group (group A, n=40) or control group (group B, n=40). Polysomnographic recordings for sleep quality assessment, Beck Depression Inventory (BDI), Profile of Mood States(POMS), Rosenberg Self-Esteem Scale (RSES), number of CD3+, CD4+, CD8+ T cells count and CD4/CD8 ratio were measured before and at the end of the study after six months.

**Results:**

There was a significant increase in the total sleep duration, sleep efficiency and sleep onset latency in group(A) after six months of aerobic exercise training, while, wake time after sleep onset and rapid eye movement (REM) latency significantly reduced after six months of aerobic training compared with values obtained prior to aerobic exercise training. Also, the mean values of BDI, POMS, CD3 count, CD4 count and CD8 count decreased significantly and the mean value of RSES significantly increased in group (A) after the aerobic exercise training, while the results of the control group were not significant. Moreover, there were significant differences between both groups at the end of the study.

**Conclusion:**

Exercise training can be considered as a non-pharmacological modalty for modifying sleep quality, psychological wellbeing and immune system among subjects with chronic primary insomnia.

## Introduction

Chronic primary insomnia is characterized by long-term difficulties in maintaining and initiating sleep, too early waking up, poor mood, fatigue, impaired concentration and poor quality of life^1^–^4^. Insomnia is a prevalent sleep disorder affecting 15%–22% of worldwide population with huge adverse impact on the general health 6–8.

Insomnia is associated with psychosocial and occupational impairments include cognitive deficits, poor mood, daytime fatigue and poor quality of life 9. Many previous studies proved an association between sleep difficulties and immune system dysfunctions among depressed subjects 10, 11; lower NK cell activity 12 and significantly reduced levels of immune cells 13.

Medication is the common line of treatment for insomnia even it is usually associated with many side effects and higher rate of mortality 14. In addition, behavioral and cognitive therapies are the common nonpharmacological interventions for insomnia treatment 15, 16. While, exercise training is another alternative treatment for insomnia which is effective and low cost 17. Many studies reported reduced severity of insomnia following exercise training 18–23.

Exercise plays a protective role against changes in the immune system that improve body efficiency for wound healing, reduction in risk for cancer and progression of tumor^24^. Exercise training interventions in previously sedentary individuals have been shown to enhance T-cell proliferative capacity 25,26.

The aim of the study was to investigate the effects of aerobic exercise training on the quality of sleep, psychological wellbeing and immune system among subjects with chronic primary insomnia.

## Patients and methods

### Subjects

**Figure (1):**
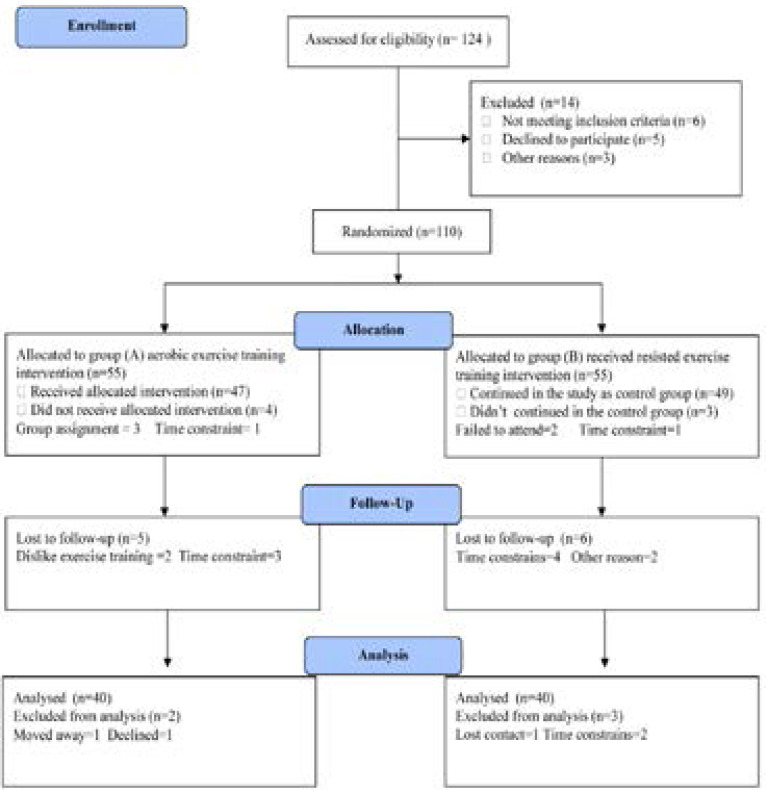
Subjects screening and recruitment CONSORT diagram.

Eighty previously sedentary subjects having chronic primary insomnia for longer than six months, their age ranged from 35–56 years and participated in this study. Exclusion criteria included history of use of psychotherapeutic drugs, shiftwork, exercise training for more than one day /week, smoking, alcohol abuse, major psychiatric disorders and caffeine intake more than 300 mg/day. Participants were enrolled in either an aerobic exercise intervention group (group A) who participated in the exercise intervention conducted three times per week for six months or non-physical activity intervention control group(group B). The CONSORT diagram outlining the details of the screening, run-in and randomization phases of the study and reasons for participant exclusion can be found in figure (1). Informed consent was obtained from all participants. This study was approved by the Scientific Research Ethical Committee, Faculty of Applied Medical Sciences at King University.

### Methods

#### Measurements

The following measurements were taken before the study and after 6 months at the end of the study.

A. Sleep measures: All participants underwent polysomnographic (PSG) recording before and after the exercise training. For the pre-intervention assessment, PSG recording was performed over 2 nights. The room used for the recordings had a large comfortable bed, acoustic isolation, and controlled temperature and light. Recordings were conducted by a trained sleep technician using a digital system (Philips-Respironics, USA)^27^.

**B. Flow cytometry analysis:** The human leukocyte differentiation antigens CD3, CD4 and CD8 (Beckman Coulter, Marseille, France) Five microliters of appropriate monoclonal antibody was added to 50 µL of a whole-blood sample and incubated for 15 minutes at room temperature. Thereafter, the erythrocytes were lysed with 125 µL of a lysing solution, OptiLyse C, for 10 minutes. The samples were analyzed by flow cytometry using Cytomics FC 500 and CXP software (Beckman Coulter).

**C. Psychological well-being:** The Rosenberg Self-Esteem Scale (RSES) was used to measure self-esteem which consisted of 10 items answered on a 4-point Likert scale. The RSES higher scores means greater self-esteem. The Profile of Mood States (POMS) was used to measure mood disturbances, POMS consists of 65 items on a 5-point Likert scale. However, the Beck Depression Inventory (BDI) was used to measure depression, which includes 21 items. The BDI higher scores mean higher depressive symptoms level 28.

### Procedures

Following the previous evaluation, all patients will be divided randomly into the following groups:

**1. The training group (Group A)** received aerobic exercise training for six months on the treadmill (Enraf Nonium, Model display panel Standard, NR 1475.801, Holand) which was conducted according to recommendation of aerobic exercise application approved by the American College of Sports Medicine 29. Training program will include 5 minutes for warming -up in the form of range motion and stretching exercises, 30 minutes of aerobic exercise training with intensity equal 60–70% of the individual maximum heart rate followed by cooling down for 10 minutes (on treadmill with low speed and without inclination). Participants had 3 sessions /week for 6 months with close supervision of physical therapist.

**2. The control group (Group B) received no exercise intervention.**

## Statistical analysis

The mean values of the investigated parameters obtained before and after six months in both groups were compared using paired “t” test. Independent “t” test was used for the comparison between the two groups (P<0.05).

## Results

The two groups were considered homogeneous regarding the demographic variables. The mean age of the group (A) was 51.27 ± 5.32 year, and the mean age of group (B) was 52.64 ± 4.81 year. There was no significant differences in age, body mass index (BMI), systolic blood pressure, diastolic blood pressure and maximum heart rate between both groups (table 1).

**Table 1:** Baseline characteristics of study participants

Characteristic	Group (A)	Group (B)	Significance
**Age** (years)	51.27 ± 5.32	52.64 ± 4.81	P > 0.05
**Gender** (male/female)	14/26	12/28	P>0.05
**BMI** (kg/m^2^)	27.13 ± 3.65	25.98 ± 3.37	P > 0.05
**SBP** (mmHg)	148.26 ± 9.14	145.75 ± 10.29	P > 0.05
**DBP** (mmHg)	87.12 ± 5.28	86.53 ± 4.76	P > 0.05
**HR_max_** (beat/min)	152.74 ± 10.91	154.35 ± 12.62	P > 0.05

BMI: Body mass index; SBP: Systolic blood pressure; DBP: Diastolic blood pressure; HR max: Maximum heart rate.

Regarding sleep quality parameter, there was a significant increase in the total sleep duration, Sleep efficiency and Sleep onset latency in group(A) after 6 months of aerobic exercise training, while, awake time after sleep onset and REM latency significantly reduced after 6 months of aerobic training compared with values obtained prior to aerobic exercise training(table 2). Moreover, the mean values of BDI, POMS, CD3 count, CD4 count and CD8 count decreased significantly and the mean value of RSES significantly increased in group (A) after the aerobic exercise training, however the results of the control group were not significant (table 3). Also, there were significant differences between both groups at the end of the study (table 4).

**Table 2:** Mean value and significance of polysomnographic parameters, psychological wellbeing parameters, CD3 count, CD4 count and CD8 count in group (A) before and after treatment

	Mean + SD		t-value	Significance
	Before	After		
**Total sleep** **duration** (min)	315.17 ± 27.26	342.54 ± 25.92*	10.13	P <0.05
**Sleep** **efficiency** (%)	69.53 ± 7.14	80.61 ± 9.38*	7.75	P <0.05
**Sleep onset** **latency** (min)	11.28 ± 3.22	14.87 ± 3.63*	6.81	P <0.05
**Awake time after** **sleep onset** (min)	79.13 ± 8.95	64.25 ± 6.42*	7.27	P <0.05
**REM sleep** **latency** (min)	88.76 ± 9.21	69.32 ± 7.11*	9.16	P <0.05
**Self-esteem (RSES)**	23.15 ± 3.29	28.26 ± 3.43*	6.51	P <0.05
**Depression (BDI)**	7.68 ± 1.54	5.17 ± 1.22*	5.25	P <0.05
**Total mood** **disturbance (POMS)**	25.36 ± 3.21	19.78 ± 2.89*	6.47	P <0.05
**CD3 count** (10^9^/L)	1.88 ± 0.92*	1.31 ± 0.83*	4.56	P<0.05
**CD4 count** (10^9^/L)	1.63 ± 0.85*	1.29 ± 0.74*	4.48	P <0.05
**CD8 count** (10^9^/L)	0.91 ± 0.26*	0.63 ± 0.18*	4.35	P <0.05

REM: rapid eye movements; RSES: Rosenberg Self-Esteem Scale; BDI: Beck Depression Inventory; POMS: Profile of Mood States

* indicates a significant difference between the two groups, P < 0.05.

**Table 3:** Mean value and significance of polysomnographic parameters, psychological wellbeing parameters,CD3 count, CD4 count and CD8 count in group (B) before and at the end of the study

	Mean + SD		t- value	Significance
	Before	After		
**Total sleep** **duration** (min)	319.36 ± 26.52	314.28 ± 27.11	1.85	P>0.05
**Sleep** **efficiency** (%)	70.14 ± 6.93	68.75± 7.13	1.54	P>0.05
**Sleep onset** **latency** (min)	12.26 ± 3.37	10.98 ± 3.12	1.35	P>0.05
**Awake time after** **sleep onset** (min)	82.32 ± 9.31	83.76 ± 9.81	1.74	P>0.05
**REM sleep** **latency (min)**	90.13 ± 10.27	92.48 ± 10.16	1.83	P>0.05
**Self-esteem (RSES)**	24.71 ± 3.45	23.26 ± 3.28	1.34	P>0.05
**Depression (BDI)**	7.35 ± 1.82	8.22 ± 1.93	1.17	P>0.05
**Total mood** **disturbance** **(POMS)**	24.21 ± 3.37	26.43± 3.75	1.36	P>0.05
**CD3 count** (10^9^/L)	1.74 ± 0.92	1.92 ± 0.97	0.85	P>0.05
**CD4 count** (10^9^/L)	1.48 ± 0.85	1.52 ± 0.86	0.78	P>0.05
**CD8 count** (10^9^/L)	0.87 ± 0.32	0.94 ± 0.35	0.67	P>0.05

REM: rapid eye movements; RSES: Rosenberg Self-Esteem Scale; BDI: Beck Depression Inventory; POMS: Profile of Mood States.

**Table 4:** Mean value and significance of polysomnographic parameters, psychological wellbeing parameters, CD3 count, CD4 count and CD8 count in group (A) and group (B) at the end of the study

	Mean + SD		t-value	Significance
	Group (A)	Group (B)		
**Total sleep** **duration** (min)	342.54 ± 25.92*	314.28 ± 27.11	8.54	P>0.05
**Sleep** **efficiency** (%)	80.61 ± 9.38*	68.75± 7.13	6.26	P <0.05
**Sleep onset** **latency** (min)	14.87 ± 3.63*	10.98 ± 3.12	5.47	P <0.05
**Awake time after** **sleep onset** (min)	64.25 ± 6.42*	83.76 ± 9.81	6.15	P <0.05
**REM sleep** **latency (min)**	69.32 ± 7.11*	92.48 ± 10.16	7.74	P <0.05
**Self-esteem (RSES)**	28.26 ± 3.43*	23.26 ± 3.28	5.21	P <0.05
**Depression (BDI)**	5.17 ± 1.22*	8.22 ± 1.93	4.63	P <0.05
**Total mood** **disturbance** **(POMS)**	19.78 ± 2.89*	26.43± 3.75	4.82	P <0.05
**CD3 count** (10^9^/L)	1.31 ± 0.83*	1.92 ± 0.97	3.61	P <0.05
**CD4 count** (10^9^/L)	1.29 ± 0.74*	1.52 ± 0.86	3.57	P <0.05
**CD8 count** (10^9^/L)	0.63 ± 0.18*	0.94 ± 0.35	3.46	P <0.05

REM: rapid eye movements; RSES: Rosenberg Self-Esteem Scale; BDI: Beck Depression Inventory; POMS: Profile of Mood States

* indicates a significant difference between the two groups, P < 0.05.

## Discussion

Insomnia has significant negative impacts on mental and physical health 30, impair quality of life, and increase healthcare costs^31^ and excessive daytime sleepiness 32. It can also impair the metabolic, endocrine, and immune systems, among other deleterious effects^33^. However, exercise promoted increased sleep efficiency and duration in populations suffering from chronic sleep complaints^34^,^35^. Concerning sleep quality parameter, the results of the present study revealed that there was a significant increase in the total sleep duration, sleep efficiency and sleep onset latency after 6 months of aerobic exercise training, while, awake time ater sleep onset and REM latency significantly reduced after 6 months of aerobic training compared with values obtained prior to aerobic exercise training in patients with chronic primary insomnia, these results are in line with many previous studies.

Reid and colleagues had Seventeen sedentary elderly subjects with insomnia who had 16 weeks of aerobic physical activity. The clearly stated that physical activity improved sleep quality on the global Pittsburgh Sleep Quality Index (PSQI) score, sleep latency, sleep duration, daytime dysfunction and sleep efficiency 36. Where, Lira et al. conducted a study on fourteen male sedentary volunteers performed moderate training for 60 minutes/day, 3 days/week for 24 weeks at a work rate equivalent to the ventilatory aerobic threshold. They proved that sleep parameters, awake time and REM sleep latency were decreased after 6 months exercise training in relation baseline values^37^. Also, Yang and colleagues completed a systematic review with meta-analysis of six randomized trials and provided data on 305 participants (241 female). Each of the studies examined an exercise training program that consisted of either moderate intensity aerobic exercise or high intensity resistance exercise. The duration of most of the training programs was between 10 and 16 weeks. All of the studies used the self-reported Pittsburgh Sleep Quality Index to assess sleep quality. Compared to the control group, the exercise group had significantly reduced sleep latency and medication use^38^. While, Chen and coworkers enrolled twenty seven participants in a 12 weeks of exercise training, they proved that overall sleep quality, subjective sleep quality, sleep latency, sleep duration, sleep efficiency, and daytime dysfunction significantly improved after 12 weeks of intervention^39^. In addition, Santos et al. had twenty-two male, sedentary volunteers performed moderate training for 60 min/day, 3 days/week for 24 week at a work rate equivalent to their ventilatory aerobic threshold, their findings suggest that aerobic exercise training increased aerobic capacity parameters, decreased REM latency and decreased time awake^40^. Moreover, Passos and colleagues concluded that 4-month intervention of moderate aerobic exercise delivered to twenty-one sedentary participants with chronic primary insomnia had polysomnographic data significantly improvements following exercise training, where total sleep time, sleep efficiency and rapid eye movements significantly increased. In addition, sleep onset latency and wake time after sleep onset significantly decreased following exercise training^41^.

Regarding, the mechanism underlying the effect of exercise on sleep, although the mechanisms by which training can improve sleep quality are not well understood. It has been proposed that exercise training improves sleep quality through increasing energy consumption, endorphin secretion, or body temperature in a manner that facilitates sleep for recuperation of the body 42–44. In addition, some other mechanisms, such as an increasing in energy consumption, endorphin secretion, body temperature, are also beneficial to improve sleep quality^45^. Moreover, moderate training may reduce resting plasma concentrations of pro-inflammatory cytokines and increase anti-inflammatory cytokines, consequently improving the quality of sleep^46^.

Our results demonstrate that aerobic exercise training led to decreased Beck Depression Inventory (BDI) & Profile of Mood States (POMS) and increased Rosenberg Self-Esteem Scale (RSES) in patients with Chronic Primary Insomnia. In this regard, some studies revealed that the aerobic exercise training has a strong impact on psychological wellbeing. Singh et al. found reductions in subjective sleep quality and depression measures in depressed subjects with insomnia after a 10-week randomized control trial of a supervised weight-training program conducted three times per week^20^. Buman and coworkers observed a decrease in depressive symptoms after a 12-month intervention of moderate aerobic exercise and demonstrated antidepressant effect of exercise is an important factor in improving sleep^47^. Passos and colleagues investigated the effects of a 6-month moderate aerobic exercise program on sleep in patientswith chronic insomnia and found a significant reduction in POMS-tension/anxiety scores as well as improvements in sleep; however, these variables did not correlate 48. Dunn et al. studied the dose response relation of exercise and reduction in depressive symptoms in patients with mild to moderate major depression who exercised individually^49^. Mota-Pereira and colleagues proved that a home-based exercise program of 30–45 min/day walks, 5 days/week for three moths improved depression and functioning parameters in treatment-resistant 150 patients with major depressive disorder^50^. Meta-analyses from 2010 by Conn included 70 studies with 2679 clinically depressed subjects and suggested that there was a moderate and statistically significant effect size for exercise in treating depression^51^. Another review conducted for the Cochrane review database, with 27 articles in total and 907 participants, showed evidence suggesting exercise was effective in the treatment of depression 52.

There are many possible explanations for the mechanisms involve improvements in self-esteem and sleep quality following exercise training, some of the main biological mechanisms are reduced production of neuro-inflammatory factors that affect the main neuro-immune mechanisms potentially leading to symptoms of depression-like behavior^53^–^55^, also release of Beta endorphins^56^, the modification in serotonin function proposed by excessive neurotrophins, especially Brain Derived Neurotrophin Factor(BDNF) 57.

A major finding of the present study proved that aerobic exercise training improved immune function among patients with chronic primary insomnia. The results of our study compatible with several previous studies suggesting exercise training promote the modulation of immune system markers. Woods et al. found that 6 months of supervised aerobic exercise training (composed of 30-minutes of brisk walking 3 times/week) in the elderly increased T-cell proliferation compared to controls in previously sedentary elderly 58. While, Kursat et al. concluded that regular and moderate aerobic exercise on treadmill for 30 minutes has favorable effects on the immune system by increasing immunoglobulines (IgA, IgG and IgM levels) which are potent protective factors^59^. Also, Buyukyazi proved that baseline NK cell percentage, and serum IgA and IgM concentrations were significantly higher among eleven elderly male athletes performing regular aerobic exercise 60. The mechanisms of exercise-induced immune changes in response to exercise training include the blood concentration of a number of stress hormones increase, including adrenaline, noradrenaline, growth hormone, β-endorphin and cortisol^61^. Moreover, another possible mechanisms of exercise immune system modulating effects include increase in the interleukin -10 (IL-10)^62^ & decreased levels of tumor necrosis factor-alpha (TNF-α), interleukin-6 (IL-6) and C-reactive protein(CRP) 63,64.

The present study has important strengths and limitations. The major strength is the supervised nature of the study. However, all exercise sessions were supervised. Moreover, the study was randomized; hence, we can extrapolate adherence to the general population. In the other hand, there were several limitations to our study. First, our sample size was relatively small, which may have limited our ability to detect differences in our primary outcomes due to low statistical power. The limited sample size also did not allow us to control for many factors that may have impacted the results, including medications, gender and race. Furthermore, the 6-month intervention may have been too short to improve other CVD risk factors and metrics related to cardiovascular function. Finally, within the limit of this study, aerobic exercise training is recommended for modifying sleep quality, psychological wellbeing and immune system among subjects with chronic primary insomnia. Additional researches with larger study populations and longer inerventions will be needed to more thoroughly assess the biochemical and cardiovascular benefits associated with exercise training in subjects with chronic primary insomnia.

## Conclusion

Exercise training can be considered as a non-pharmacological modality for modifying sleep quality, psychological wellbeing and immune system among subjects with chronic primary insomnia.

## References

[1] Blay SL, Baxter AS, Leite GF (2008). Prevalence of self-reported sleep disturbance among older adults and the association of disturbed sleep with service demand and medical conditions. International psychogeriatrics.

[2] Wong WS, Fielding R (2011). Prevalence of insomnia among Chinese adults in Hong Kong: a population-based study. Journal of Sleep Research.

[3] Ancoli-Israel S, Ayalon L, Salzman C (2008). Sleep in the elderly: normal variations and common sleep disorders. Harvard Review of Psychiatry.

[4] Ayalon L, Liu L, Ancoli-Israel S (2004). Diagnosing and treating sleep disorders in the older adult. The Medical Clinics of North America.

[5] Dam TT, Ewing S, Ancoli-Israel S, Ensrud K, Redline S, Stone K (2008). Association between sleep and physical function in older men: the osteoporotic fractures in men sleep study. J Am Geriatr Soc.

[6] Sansoni P, Vescovini R, Fagnoni F, Biasini C, Zanni F, Zanlari L (2008). The immune system in extreme longevity. Exp Gerontol.

[7] Chasens ER, Sereika SM, Weaver TE, Umlauf MG (2007). Daytime sleepiness, exercise, and physical function in older adults. J Sleep Res.

[8] Colbert LH, Visser M, Simonsick EM, Tracy RP, Newman AB, Kritchevsky SB (2004). Physical activity, exercise, and inflammatory markers in older adults: findings from the health, aging and body composition study. J Am Geriatr Soc.

[9] Murasko DM, Goonewardene IM (1990). T-cell function in aging: mechanisms of decline. Annu Rev Gerontol Geriat.

[10] Gozal D (2009). Sleep, sleep disorders and inflammation in children. Sleep Med.

[11] Kapsimalis F, Basta M, Varouchakis G, Gourgoulianis K, Vgonzas A, Kryger M (2008). Cytokines and pathological sleep. Sleep Med.

[12] Krueger JM (2008). The role of cytokines in sleep regulation. Curr Pharm Des.

[13] Santos RV, Tufik S, de Mello MT (2007). Exercise, sleep and cytokines: is there a relation?. Sleep Med Rev.

[14] Weinert BT, Timiras PS (2003). Theories of aging. J Appl Physiol.

[15] Chandra RK (2003). Nutrient regulation of immune functions. Forum Nutr.

[16] Franceschi C, Capri M, Monti D, Giunta S, Olivieri F, Sevini F, Panourgia MP, Invidia L, Celani L, Scurti M, Cevenini E, Castellani GC, Salvioli S (2007). Inflammaging and anti-inflammaging: a systemic perspective on aging and longevity emerged from studies in humans. Mech. Ageing Dev.

[17] Pedersen M, Bruunsgaard H, Weis N, Hendel HW, Andreassen BU, Eldrup E, Dela F, Pedersen BK (2003). Circulating levels of TNF-alpha and IL-6-relation to truncal fat mass and muscle mass in healthy elderly individuals and in patients with type-2 diabetes. Mech. Ageing Dev.

[18] King AC, Pruitt LA, Woo S, Castro CM, Ahn DK, Vitiello MV, Woodward SH, Bliwise DL (2008). Effects of moderate-intensity exercise on polysomnographic and subjective sleep quality in older adults with mild to moderate sleep complaints. Journals of Gerontology Series A: Biological Sciences and Medical Sciences.

[19] King AC, Oman RF, Brassington GS, Bliwise DL, Haskell WL (1997). Moderate-intensity exercise and self-rated quality of sleep in older adults. A randomized controlled trial. JAMA.

[20] Singh NA, Clements KM, Fiatarone MA (1997). A randomized controlled trial of the effect of exercise on sleep. Sleep.

[21] Trinder J, Paxton SJ, Montgomery I, Fraser G (1985). Endurance as opposed to power training: their effect on sleep. Psychophysiology.

[22] Kubitz KA, Landers DM, Petruzzello SJ, Han M (1996). The effects of acute and chronic exercise on sleep. A meta-analytic review. Sports Med.

[23] Dolezal BA, Neufeld EV, Boland DM, Martin JL, Cooper CB (2017). Interrelationship between Sleep and Exercise: A Systematic Review. Adv Prev Med.

[24] Driver HS, Taylor SR (2000). Exercise and sleep. Sleep Med Rev.

[25] Youngstedt SD (2005). Effects of exercise on sleep. Clin Sports Med.

[26] Edinger JD, Morey MC, Sullivan RJ, Higginbotham MB, Marsh GR, Dailey DS (1993). Aerobic fitness, acute exercise and sleep in older men. Sleep.

[27] Rechtschaffen A, Kales AA (1968). Manual of standardized terminology, techniques, and scoring system for sleep stages of human subjects.

[28] Palmeira A, Branco T, Martins S, Minderico C, Silva M, Vieira P (2010). Change in body image and psychological well-being during behavioral obesity treatment: Associations with weight loss and maintenance. Body Image.

[29] American College of Sports Medicine (2005). Guidelines for graded exercise testing and exercise prescription.

[30] Gleeson M, Bishop N.C, Stensel D.J, Lindley M.R, Mastana S.S, Nimmo M.A (2011). The anti-inflammatory effects of exercise: mechanisms and implications for the prevention and treatment of disease. Nat. Rev. Immunol.

[31] Morgan K (2003). Daytime activity and risk factors for late-life insomnia. Journal of Sleep Research.

[32] Chen K.-M, Chen M.-H, Chao H.-C, Hung H.-M, Lin H.-S, Li C.-H (2009). Sleep quality, depression state, and health status of older adults after silver yoga exercises: cluster randomized trial. International Journal of Nursing Studies.

[33] Irwin M.R, Olmstead R, Motivala S.J (2008). Improving sleep quality in older adults with moderate sleep complaints: a randomized controlled trial of Tai Chi Chih. Sleep.

[34] Dolezal BA, Neufeld EV, Boland DM, Martin JL, Cooper CB (2017). Interrelationship between Sleep and Exercise: A Systematic Review. Adv Prev Med.

[35] Erlacher C, Erlacher D, Schredl M (2015). The effects of exercise on self-rated sleep among adults with chronic sleep complaints. Journal of Sport and Health Science.

[36] Reid KJ, Baron KG, Lu B, Naylor E, Wolfe L, Zee PC (2010). Aerobic exercise improves self-reported sleep and quality of life in older adults with insomnia. Sleep Med.

[37] Lira FS, Pimentel GD, Santos RV, Oyama LM, Damaso AR, Oller do Nascimento CM, Viana VA, Boscolo RA, Grassmann V, Santana MG, Esteves AM, Tufik S, de Mello MT (2011). Exercise training improves sleep pattern and metabolic profile in elderly people in a time-dependent manner. Lipids Health Dis.

[38] Yang PY, Ho KH, Chen HC, Chien MY (2012). Exercise training improves sleep quality in middle-aged and older adults with sleep problems: a systematic review. J Physiother.

[39] Chen M, Liu H, Huang H, Chiou A (2012). The effect of a simple traditional exercise programme (Baduanjin exercise) on sleep quality of older adults: A randomized controlled trial. International Journal of Nursing Studies.

[40] Santos RV, Viana VA, Boscolo RA, Marques VG, Santana MG, Lira FS, Tufik S, de Mello MT (2012). Moderate exercise training modulates cytokine profile and sleep in elderly people. Cytokine.

[41] Passos GS, Poyares D, Santana MG, Teixeira AA, Lira FS, Youngstedt SD, dos Santos RV, Tufik S, de Mello MT (2014). Exercise Improves Immune Function, Antidepressive Response, and Sleep Quality in Patients with Chronic Primary Insomnia. Biomed Res Int.

[42] Horne JA, Moore VJ (1985). Sleep EEG effects of exercise with and without additional body cooling. Electroencephalography and Clinical Neurophysiology.

[43] Driver HS, Taylor SR (2000). Exercise and sleep. Sleep Medicine Reviews.

[44] Li F, Fisher KJ, Harmer P, Irbe D, Tearse RG, Weimer CJ (2004). Tai chi and self-rated quality of sleep and daytime sleepiness in older adults: a randomized controlled trial. Journal of American Geriatrics Society.

[45] Yang PY, Ho KH, Chen HC, Chien MY (2012). Exercise training improves sleep quality in middle-aged and older adults with sleep problems: a systematic review. J. Physiother.

[46] Kapsimalis F, Basta M, Varouchakis G, Gourgoulianis K, Vgonzas A, Kryger M (2008). Cytokines and pathological sleep. Sleep Med.

[47] Buman MP, Hekler EB, Bliwise DL, King AC (2011). Moderators and mediators of exercise-induced objective sleep improvements in midlife and older adults with sleep complaints. Health Psychol.

[48] Passos GS, Poyares D, Santana MG, D'Aurea CVR, Youngstedt SD, Tufik S (2011). The effects of moderate aerobic exercise training on chronic primary insomnia. Sleep Med.

[49] Dunn A, Trivedi M, Kampert J, Clark C, Chambliss H (2005). Exercise treatment for depression: Efficacy and dose response. Am J Prev Med.

[50] Mota-Pereira J, Silverio J, Carvalho S, Ribeiro JC, Fonte D, Ramos J (2011). Moderate exercise improves depression parameters in treatment-resistant patients with major depressive disorder. J Psychiatr Res.

[51] Conn V (2010). Depressive symptom outcomes of physical activity interventions: meta-analysis findings. Ann. Behav. Med.

[52] Mead GE, Morley W, Campbell P, Greig CA, McMurdo M, Lawlor DA (2009). Exercise for depression. Cochrane Database Syst Rev.

[53] Kubera M, Obuchowicz E, Goehler L, Brzeszcz J, Maes M (2011). In animal models, psychosocial stress-induced (neuro)inflammation, apoptosis and reduced neurogenesis are associated to the onset of depression. Prog. Prog Neuropsychopharmacol Biol Psychiatry.

[54] Wager-Smith K, Markou A (2011). Depression: a repair response to stress-induced neuronal microdamage that can grade into a chronic neuroinflammatory condition?. Neurosci Biobehav Rev.

[55] Capuro L, Miller AH (2011). Immune system to brain signaling: neuropsychopharmacological implications. Pharmacol. Ther.

[56] Dishman R, O'Connor P (2009). Lessons in exercise neurobiology: the case of endorphins. Mental Health and Physical Activity.

[57] Lucassen P, Meerlo P, Naylor A, Van Dam A, Dayer A, Fuchs E, Oomen C, Czeh B (2010). Regulation of adult neurogenesis by stress, sleep disruption, exercise and inflammation: implications for depression and antidepressant action. European Neuropsychopharmacology.

[58] Woods JA, Ceddia MA, Wolters BW, Evans JK, Lu Q, McAuley E (199). Effects of 6 months of moderate aerobic exercise training on immune function in the elderly. Mech. Ageing Dev.

[59] Karacabey Kursat, Saygin Ozcan, Ozmerdivenli1 Recep, Zorba2 Erdal, Godekmerdan Ahmet, Bulut Vedat (2005). The effects of exercise on the immune system and stress hormones in sportswomen. Neuroendocrinol Lett.

[60] Buyukyazi G (2004). Differences in the cellular and humoral immune system between sedentary and endurance-trained elderly males. Science & Sports.

[61] Marsland A.L, Herbert T.B, Muldoon M.F, Bachen E.A, Patterson S, Cohen S, Rabin B, Manuck S.B (1997). Lymphocyte subset redistribution during acute laboratory stress in young adults: mediating effects of hemoconcentration. Health Psychology.

[62] Jankord R, Jemiolo B (2004). Influence of physical activity on serumIL-6 and IL-10 levels in healthy older men. Medicine and Science in Sports and Exercise.

[63] Bachen E.A, Manuck S.B, Cohen S, Muldoon M.F, Raible R, Herbert T, Rabin B.S (1995). Adrenergic blockade ameliorates cellular immune responses to mental stress in humans. Psychosomatic Medicine.

[64] Starkie R, Ostrowski S, Jauffred S, Febbraio M, Pedersen B (2003). Exercise and IL-6 infusion inhibit endotoxin induced TNF-alpha production in humans. The FASEB Journal.

